# The safety and efficacy of neuromodulation using percutaneous electrical nerve stimulation for the management of trigeminal‐mediated headshaking in 168 horses

**DOI:** 10.1111/evj.13174

**Published:** 2019-09-23

**Authors:** V. L. H. Roberts, M. Bailey, The Equipens™ group, H. B. Carslake, F. Malalana, C. McGowan, E. F. Haggett, T. Barnett, D. I. Rendle, U. Lunden, A. R. Fiske‐Jackson, V. E. South, J. Prutton, A. Durham, R. Findshøj, H. Panhuijzen, R. Van Der Rijt, T. Booth, M. Robin, A. G. Raftery, K. J. Pickles, N. K. Patel

**Affiliations:** ^1^ Bristol Veterinary School University of Bristol Somerset UK; ^2^ Institute of Neurosciences Southmead Hospital Bristol Westbury‐on‐Trym, Bristol UK

**Keywords:** horse, headshaking, trigeminal‐mediated, neuromodulation, percutaneous electrical nerve stimulation

## Abstract

**Background:**

Early results from the use of neuromodulation by percutaneous electrical nerve stimulation for the management of trigeminal‐mediated headshaking in horses were promising but lacked sufficient case numbers and long‐term follow‐up. The neuromodulatory procedure has since been established as EquiPENS™.

**Objectives:**

The aim of this study was to report long‐term results from a larger number of cases and to investigate for predictors of outcome.

**Study design:**

Prospective case series using international, multi‐centre data.

**Methods:**

Eligible cases were horses with a veterinary diagnosis of trigeminal‐mediated headshaking, which received EquiPENS™ neuromodulation at trained centres between August 2013 and November 2017. The standard protocol was an initial three‐procedure course, with additional procedures should a horse go into remission but then relapse. Data collected included signalment, history, diagnostic tests performed, details of any complications, whether horses had gone into remission and the length of remission.

**Results:**

Results were obtained from 168 horses, with 530 procedures. The complication rate was 8.8% of procedures. In all but one case, complications were mild and transient, without self‐trauma. Remission of headshaking following the initial course occurred in 53% (72/136) of horses. Median length of time recorded in remission was 9.5 weeks (range 2 days to 156 weeks ongoing). Where signs recurred, most horses went back into remission following additional procedures, usually for longer than from the previous procedure. No predictors for outcome were determined.

**Main limitations:**

No placebo or control group, owner‐assessed results.

**Conclusions:**

EquiPENS™ neuromodulation can be an effective and safe treatment for the management of trigeminal‐mediated headshaking in some horses. An increased understanding of neuromodulation could help optimise the technique. Advances in treatment for trigeminal‐mediated headshaking will remain limited until there is a greater understanding of the aetiopathogenesis of the condition.

## Introduction

Trigeminal‐mediated headshaking is an idiopathic neuropathic facial pain condition of the horse. The condition can have important welfare implications 1. It carries a poor prognosis, with published treatments lacking consistent efficacy, and in some instances, safety and practical application 2, 3, 4, 5. The use of a nose‐net improves clinical signs by 70% or greater in one‐third of horses 6. A nose‐net is non‐invasive and cheap and is usually the first‐line of treatment but its use in competition is not accepted by all equestrian organisations. Medical treatment with carbamazepine and/or cyproheptadine 2, 7 can be effective in some individuals. However, in general, results are inconsistent, and the use of these pharmaceuticals can be expensive, associated with side effects of drowsiness and would not usually be permitted in competition. The surgical placement of platinum coils within the infraorbital canal was reported to have a 49% remission rate 5 but resulted in adverse effects of enough severity as to result in euthanasia in 4/58 horses.

Advances in the treatment for trigeminal‐mediated headshaking will remain limited until the aetiopathogenesis of the condition is understood. The most recent advance was that the trigeminal nerve of affected horses is sensitised, having a reduced threshold for activation, which is thought to result in neuropathic pain 8. The nerve is functionally abnormal but appears to be structurally normal 9 which could mean that the sensitisation is reversible. Following on from this, percutaneous electrical nerve stimulation, a repeatable, minimally invasive neuromodulatory therapy with some application for the management of neuropathic pain in people, was trialled in seven horses 10. All horses tolerated the procedure well. Three horses developed a haematoma at the site on one occasion and two had increased clinical signs suspected to be due to a transient neuritis, for up to 3 days following one of the procedures. All horses underwent a minimum of three procedures, with two horses having four procedures. Following their last procedure, remission, classed as ridden work at their previous level, was obtained in five horses, with a median remission time of 15.5 weeks from the third procedure and 20 weeks from the fourth. These early results were promising for management of the condition but lacked sufficient case numbers and long‐term follow‐up. Following this, the use of electroacupuncture, which uses a different electrical algorithm, equipment and technique, gave positive results in a small number of suspected trigeminal‐mediated headshakers 11.

The percutaneous electrical neuromodulatory procedure has since been established as EquiPENS™ and various centres across the UK and Europe have been trained by the first author in the technique, using the same equipment^1^, ensuring consistency. The aim of this study is to report long‐term results for treatment with EquiPENS™ and to identify any predictors for outcome from a larger number of cases, using international, multi‐centre data.

## Materials and methods

Eligible cases were horses with a veterinary diagnosis of trigeminal‐mediated headshaking, which received EquiPENS™ neuromodulation at trained centres between August 2013 and November 2017. Centres were issued with guidelines for reaching a diagnosis of trigeminal‐mediated headshaking. These are detailed in Supplementary Item 2. Horses could have previously received, but responded insufficiently to, other treatments for headshaking, but were not receiving any other treatment at the time of enrolment. In order to assess response to treatment, horses were in ridden or lunge work prior to the onset of clinical signs of headshaking. EquiPENS™ neuromodulation was performed as described previously 10. The protocol was for each horse to receive an initial course of three procedures. The second procedure was performed 3–7 days after the first procedure, and the third procedure was performed 10–14 days after the second procedure. Intervals were extended if a horse went into remission after a procedure, until it relapsed. If the horse went into remission after three procedures but then relapsed, additional procedures could be performed.

A standard recording form was available to each centre for prospective collection of data regarding each horse’s signalment, history and the diagnostic tests they performed. The information requested included whether signs were known to be spring/summer seasonal only or present all year round. Seasonality of signs was recorded as unknown in those cases which were reported to have been affected for less than a year. The diagnosis of trigeminal‐mediated headshaking was made by exclusion. If during investigation abnormalities were detected, these were judged to not be clinically relevant, sometimes after treatment with no response. Examples would include sharp dental arcades and iris cyst.

Centres were also asked to record the following information for each EquiPENS™ procedure performed for each case: the date of the procedure; whether any complications were experienced during or after the procedure and the nature of those complications; whether the horse went into remission; and if so, for how long. Remission was defined as return to ridden exercise (or if not trained, lunge exercise) at, or above, the level of performance prior to the onset of clinical signs, within 3 weeks from the last procedure. This was an assessment made by the owner. Follow‐up on each case was obtained by the centre responsible for that case by telephone questionnaire (Supplementary Item 3). The longest‐term available follow‐up was used in this study. Where horses had been initially classified as going into remission with an initial follow‐up performed prior to March 2016, follow‐up was repeated between July and November 2017. Where horses could no longer be classified as being in remission, whether this was due to recurrence of headshaking signs or because horses could no longer be assessed as they were out of ridden work for other reasons, was recorded. Otherwise, remission was recorded as being on‐going at the time of follow‐up.

### Data analysis

Statistical analysis was performed using the *survival* and *survminer* packages for the R statistical program (Version 3.4.4)^2^ and R Studio (Version 1.1.423)^b^ running on Ubuntu 17.10^3^. Censored survival objects were created using the *Surv* function of the survival package and Kaplan Meier plots created using the *survfit* and *ggsurvplot* functions. Probabilities for differences in length of remission after third or final treatment dependent on response to first or second treatments were calculated using log‐rank methods. Cox proportional hazards for presence of and differences in length of remission dependent on management, centre and history prior to treatment were calculated using the *coxph* function. Variables tested were: the time for which the horse had been affected with clinical signs prior to presentation; whether use of a nosenet had, judged by the owner, no effect on clinical signs, insufficient effect or aggravated clinical signs; whether diagnostic local anaesthesia had been performed, the location of that anaesthesia and the result; seasonality.

## Results

### Population statistics

There were 168 horses fitting the inclusion criteria. The median age at presentation was 9 years (range 2–21 years, n = 163, 5 not recorded). The most common groups of breeds were sports horses (49/160, 31% 8 not recorded) and warmbloods (42/160, 26%). Age ranges appeared evenly distributed across the breeds (Supplementary Item 4). There were 120/165 (73%) geldings, 42/165 (25%) mares and 3/165 (2%) stallions. The sex of 3 horses was not recorded. Procedures were performed at 13 trained centres across the UK and Europe. On occasion, diagnostics and the first one or two procedures were performed at one centre, with other procedures performed at a different centre. In these instances, the first centre only was attributed the case. The distribution of cases across the centres is displayed in Supplementary Item 5.

Most horses (94/166, 57%, 2 not recorded) were used for general riding. Distribution of use is displayed in Supplementary Item 6.

### Prior treatment

Nose‐nets had been trialled for treatment in 128 horses and it was reported by the owner to have no, or insufficient, effect in 109/128 (84%) cases and worsen clinical signs in 16/128 (12%) cases. Three horses whose clinical signs were alleviated by use of a nose‐net were presented for treatment because they were unable to compete in their discipline with a nose‐net.

Distribution of the main other attempted treatments are shown in Supplementary Item 7. None of these treatments were reported by the owners to have been effective or sufficiently effective. Less commonly used, attempted but failed, treatments reported by owners are detailed in Supplementary Item 8.

### Diagnosis

All horses were diagnosed with trigeminal‐mediated headshaking by veterinary surgeons. European or American board‐certified specialists were responsible for case management at 9/13 centres. The median length of time for which horses were affected prior to presentation was 4 months (range 0.5–84 months, n = 153, 15 not recorded). There were 21/158 (13% 10 not recorded) horses known to be Spring/Summer seasonally affected and 62/158 (39%) horses known to be affected all year round. Seasonality was unknown i.e. horses having been affected for less than a year, in 75/158 cases (48%).

The diagnostic techniques performed included computed tomography of the head in 124/168 (85%) cases (21 not performed, 23 not recorded). Diagnostic local anaesthesia was performed bilaterally at the caudal point of the infraorbital canal in 51 cases, giving a positive result in 35/51 (68%). Bilateral diagnostic local anaesthesia of the infraorbital nerve at the level of the infraorbital foramen was performed in a further three cases, giving a positive result in two cases. Diagnostic local analgesia was not performed in 79/133 (59%) cases)**.**

### Procedure information

A total of 530 procedures were performed. Of the 168 horses which began the initial three‐procedure course, 156 horses had completed the course at the time of follow up. Details regarding non‐completion are in Table 1.

**Table 1:** Reasons for horses enrolled in the study to have not completed the initial three‐procedure course at the time of follow‐up

		Reason for non‐completion					
Procedure number	Number of horses	In remission	Withdrawn due to developing other medical condition	Horse compliance	Owner compliance	Course not yet completed at time of follow up	Unknown
1	168						
2	165			2		1	
3	156	2	1	1	1	3	1

If horses had gone into remission following the initial three‐procedure course, they were eligible for an additional procedure should signs return. Of these eligible horses, 26 underwent a fourth procedure, 11 underwent a fifth procedure and 4 underwent a sixth procedure.

The median interval between the first and second procedures was 6 days (range 3–36 days due to long remission from the first procedure, n = 133, interval not recorded in 32 horses, three horses did not continue to a second procedure or had not done so by the time of follow‐up (Table 1) and the second and third was 14 days (range 4–165 days due to long remission from the second procedure, n = 125, interval not recorded in 31 horses, 9 horses did not continue to a third procedure or had not done so by the time of follow‐up (Table 1).

### Complications

Complications were reported in 47/530 procedures (8.8%, Table 2). In all but one case, complications were transient. Of these, worsening of signs presumed to be secondary to neuritis was the most frequent complication occurring in 18/530 (3.4% procedures). No self‐trauma was reported. All but one horse’s signs returned at least to pre‐treatment baseline in a few days and this occurred more rapidly if dexamethasone was administered intravenously at 0.1 mg/kg. One horse, treated soon after the onset of headshaking, showed a worsening of signs after each of two procedures. The horse’s compliance was also poor, so it was decided not to perform the third procedure and that horse’s clinical signs continued to progress. It is unclear whether this progression was a direct result of the neuromodulatory therapy, or the natural disease course in this horse. Suspected neuritis was reported more than once over the treatment course in four cases, which accounted for 9/18 (50%) of episodes. This was reported on two occasions in three cases; in one case with the first and third procedures and in the others with the first and second. One of these is the horse detailed above which had also complied poorly with the procedures, so a third procedure was not attempted. In the other case, dexamethasone was given prior to the third procedure and no subsequent neuritis was observed. Suspected neuritis occurred after all three procedures in one case.

**Table 2:** Complications recorded and numbers affected for each procedure

	Complication					
Procedure number	Suspected neuritis	Horse compliance	Haematoma	Dermatitis	Catheter complication	Colic
1	7	2	6	4	3	1
2	6	1	4	2	0	0
3	5	0	5	0	0	0
4	0	0	0	0	0	0
5	0	0	1	0	0	0
6	0	0	0	0	0	0

### Success rates

**Figure 1:**
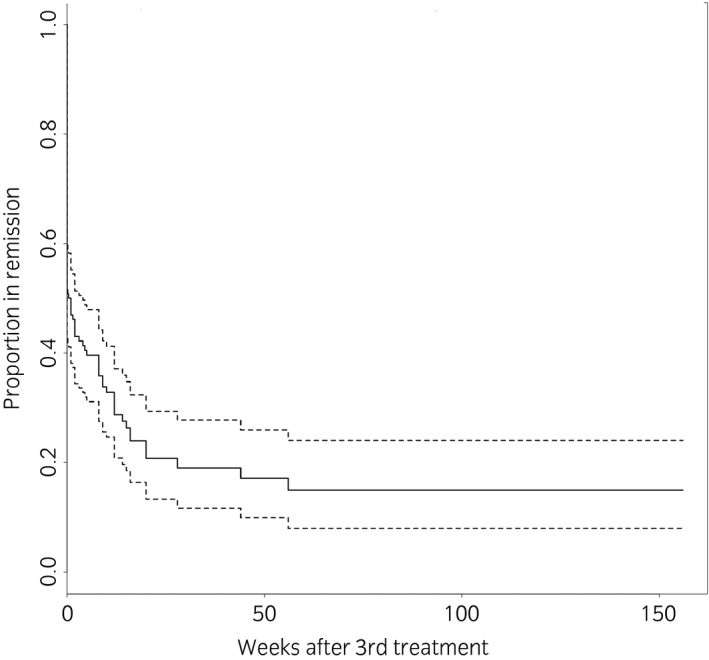
Kaplan‐Meier survival plot demonstrating the proportion of horses entering remission at the end of the initial three‐procedure course and the maintenance of remission over time. The dotted lines are 95% confidence intervals.

The proportion of horses going into remission following the initial three‐procedure course was 53% (72/136 with follow‐up data which includes two horses which remained in remission after two procedures and did not complete the third, 64/136 were non‐responders, 20/156 were lost to follow‐up or could not be evaluated due to being out of work because of other conditions). The median duration of remission was 9.5 weeks (range 2 days to 156 weeks and ongoing). This calculation of the duration of remission included not only the length of time until signs returned (36/72, 50%) but also the time to follow‐up for those still in remission at the end of the study (33/72, 46%). There were a small number of horses (3/72, 4%) which, having gone into remission were then out of work for reasons other than headshaking. For these horses, the duration of remission was taken to be until the last date the horses could be assessed i.e. when still in ridden work. Figure 1 displays how remission was sustained over time. At 56 weeks following the third procedure, 15% of cases remained in remission and these then remained in on‐going remission at the last follow up at 156 weeks, with no further horses relapsing.

Within the initial three‐procedure course, 35% (46/130) of horses went into remission after the first procedure (84/130 not in remission, 38/168 not recorded) with a median length of remission of 3 days (range, 1–14 days) before relapsing prior to the second procedure. Following the second procedure, 43% (60/137) of horses went into remission (77/137 not in remission, 28/165 not recorded) before relapsing prior to the third procedure. The median length of remission was 2 weeks (range, 2 days to 61 weeks) before relapse.

A Cox proportional hazards regression model was performed to assess for significant predictors of remission after the initial three‐procedure course. Nerve blocks, centre and seasonality were all non‐significant (P>=0.15) and are displayed in Table 3. However, horses were significantly (P<0.001) more likely to go into remission at the end of the first three‐procedure course if they went into remission after the first procedure and second procedures, but horses could still respond if the first procedure was unsuccessful.

**Table 3:** Results of Cox proportional hazards regression model for predictors from history on outcome of remission or not after the initial three procedure course

Variable	Coef	Exp (coef)	Se (coef)	z	P
Duration of clinical signs prior to presentation	−2.322e−02	9.770e−01	1.700e−02	−1.366	0.2
Caudal infra‐orbital nerve block, negative result	4.373e−01	1.549e+00	4.936e−01	0.886	0.4
Rostral in infra‐orbital nerve block, negative result	−1.723e+01	3.288e−08	4.143e+03	−0.004	>0.9
Caudal infra‐orbital nerve block, positive result	2.777e−01	1.320e+00	3.469e−01	0.800	0.4
Rostral in infra‐orbital nerve block, positive result	1.906e+00	6.724e+00	1.139e+00	1.673	0.09
Nosenet – no response	−1.361e+00	2.565e−01	1.149e+00	−1.185	0.2
Nosenet – insufficient response	−1.551e+00	2.121e−01	1.183e+00	−1.311	0.2
Nosenet – worse signs	−1.423e+00	2.409e−01	1.219e+00	−1.167	0.2
Seasonal signs	−8.198e−02	9.213e−01	5.636e−01	−0.145	0.9
Seasonality unknown	−7.643e−02	9.264e−01	3.500e−01	−0.218	0.8
Centre 2	−8.944e−01	4.088e−01	1.047e+00	−0.854	0.4
Centre 3	8.620e−01	2.368e+00	8.560e−01	1.007	0.3
Centre 4	−4.572e−03	9.954e−01	5.147e−01	−0.009	>0.9
Centre 5	1.323e−01	1.141e+00	6.398e−01	0.207	0.8
Centre 6	7.549e−02	1.078e+00	1.043e+00	0.072	0.9
Centre 7	NA	NA	0.000e+00	NA	NA
Centre 8	NA	NA	0.000e+00	NA	NA
Centre 9	2.912e−01	1.338e+00	6.403e−01	0.455	0.6
Centre 10	1.007e−01	1.106e+00	1.062e+00	0.095	0.9
Centre 11	−1.023e+00	3.596e−01	7.896e−01	−1.295	0.2
Centre 12	1.142e+00	3.132e+00	7.822e−01	1.460	0.1
Centre 13	2.298e−01	1.258e+00	6.207e−01	0.370	0.7

There were 26 horses which, following recurrence after remission from the initial course, received a fourth procedure. Of these initial responders, 69% (18/26) went back into remission. There were 32% of horses still in remission 12 weeks after the fourth procedure and these remained in on‐going remission with up to 140 weeks’ follow‐up.

**Figure 2:**
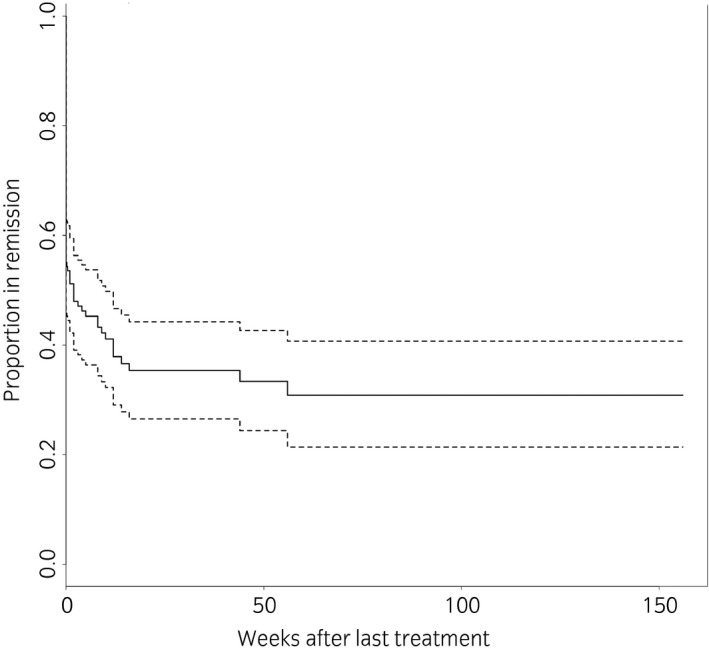
Kaplan‐Meier survival plot demonstrating the proportion of horses entering remission after their last procedure and the maintenance of remission over time. The dotted lines are 95% confidence intervals.

There were 11 horses which received a fifth procedure. Of these, 82% (9/11) went back into remission. There were 30% of these horses still in remission 14 weeks after the fifth procedure and these horses remained in on‐going remission with up to 140 weeks’ follow‐up. Figure 2 demonstrates the proportion of horses in remission after the last procedure they received and the maintenance of remission over time. There were four horses which went on to have a sixth procedure following recurrence of signs after a fifth procedure. Two went back into remission, which was ongoing at 28 and 56 weeks after this sixth procedure.

## Discussion

This study demonstrates that EquiPENS™ neuromodulation can be an effective and safe treatment for the management of trigeminal‐mediated headshaking in some horses. The data suggest that approximately 50% of cases will respond to the initial three‐procedure course. Crucial, however, is whether remission is sustained and if not, how long until signs can be expected to recur and whether further treatment will result in remission again. Our results suggest that should a horse remain in remission for longer than 56 weeks from the initial course, the remission might last for years. However, for most horses, it can be expected that remission will be shorter. The median length of remission was 9.5 weeks. However, it should be noted that almost half of the cases were still in remission at the time of follow‐up, so a longer study would be likely to show a longer median remission time. When signs recur, most but not all, horses can be expected to return to remission following additional procedures. This return to remission was usually but not always longer‐lasting than from after the previous procedure.

There was wide individual variation in how horses responded and no predictors of response were identified. Whilst horses were more likely to go into remission at the end of the initial three‐procedure course if they had gone into remission following the first and second procedures, this was not exclusively the case. The only way to know if a horse will respond is to attempt treatment. Where horses responded to the initial course it was not possible to predict length of remission. Although most horses which responded but then relapsed did respond again to additional procedures, this was not the case for all. Even in a successful case, this uncertainty would impact on a horse’s re‐sale value.

The numbers of horses going into remission increased with each subsequent treatment within the initial course. Horses were more likely to go into remission at the end of the course if they had responded during the course. This suggests a minimum of three procedures should be undertaken. There may be benefit in continuing to pursue treatment in horses which have not responded to the third procedure, but with reducing odds of success. It is, therefore, the authors’ recommendation that the protocol of performing an initial three‐procedure course be continued with a fourth procedure only being offered to responders which have then relapsed.

A further advantage of EquiPENS™ is the short competition detection time for pharmaceuticals used during the procedure, namely sedation and local anaesthetic. This allowed several horses in this study to compete at a high level whilst their clinical signs were managed with EquiPENS™.

Studies of headshaking are at risk of a placebo effect and false positive results due to spontaneous remission. There are two studies on headshaking treatments which were designed in such a way as to be able to quantify the placebo effect on owners of treatments for this condition 12, 13. The study by Pickles *et al*. 12 investigated an effect of a gonadotrophin‐releasing hormone vaccine in trigeminal‐mediated headshakers. One‐third of owners reported a subjective improvement in headshaking signs, but no real improvement was detected when considering results of serial scoring of signs on a visual analogue scale made by the same owners. The study by Talbot *et al*. 13 was a double‐blinded placebo‐controlled trial of a commercially available feed supplement for headshaking. Videos taken at set time‐points within the study were assessed by veterinary surgeons with no significant difference found across the study, contrasting with the owners’ positive subjective impression of effect on headshaking signs of both the supplement and the placebo**.** Ideally, our study would have included a placebo or control group. The data from our study suggests that for a control to be valid, the horse should be monitored for clinical signs for 1 year. During this year, no other treatments could be administered and the horse not euthanased. It would not be ethical to enrol horses into this study to leave them without treatment for a painful condition. A sham treatment course could be used, inserting the probes under sedation but not performing electrical stimulation. The sham treatment would have to be performed first and the real treatment 6 weeks later. Government ethical approval would have to be obtained for this study and it would be unlikely to be obtained, given the considerable time‐period where no other treatment could be administered if horses were showing clinical signs of pain. The results of this study, relying on owner reports at follow‐up, must be considered in the light of a lack of a placebo or control group. However, steps were taken in study design to reduce a placebo effect. All horses were showing clinical signs at the time of treatment. Remission was taken as return to ridden exercise at the previous level, in all but one case which was not yet in ridden work and so was assessed on the lunge, within 3 weeks of the last procedure.

The practical nature of the definition given to remission was chosen as being less likely to result in a false positive result than the owners’ subjective interpretation of improvement in signs 12, 13.

There is a risk of false positive results, with headshaking signs having resolved due to a change of season, or to spontaneous resolution of the condition. To reduce this risk, signs had to resolve within 3 weeks of the last procedure for that resolution to be attributed to the neuromodulation. There was no effect of seasonality on chances of, or length of, remission. Spontaneous remission, considered to be where signs resolved for 1 year or greater, to differentiate from seasonal remission, is reported from an owner survey to occur in 5% of headshakers 3. The median length of time for which horses were affected prior to presentation was 4 months, range 0.5‐84 months. It would seem unlikely that the 50% of horses going into remission within 3 weeks of neuromodulation would all have recovered spontaneously.

It is possible that some of the horses in this study were mis‐diagnosed, which would confound results. The diagnosis of trigeminal‐mediated headshaking is currently one of exclusion, making mis‐diagnosis more likely than for a condition where there is a specific test. Furthermore, the diagnoses were made by several different veterinary surgeons at different centres and in different countries. All centres were provided with guidelines for reaching a diagnosis. These guidelines had to accommodate some variation between centres, for example computed tomography of the head was not available at the time in all the countries. It may be possible in the future to further develop a technique measuring somatosensory evoked potentials as a definitive test for trigeminal nerve sensitisation, which alongside the guidelines issued to centres, would improve accuracy of diagnosis.

Complication rates were low, and in all but one case, all side‐effects were transient. This is of importance for a technique which must be repeated and has far from a guarantee of success. Where horses exhibited signs consistent with suspected neuritis, there was a strong tendency for these signs to occur again following the next procedure. In these cases, there may be some merit to considering administration of dexamethasone prior to the next procedure. This may reduce the risk of recurrence of suspected neuritis, although this is based on only one horse. There may also be merit to local administration of corticosteroid, although the authors have not trialled this yet. Even in horses where dexamethasone was not administered for suspected neuritis, signs did not result in self‐trauma and resolved in a few days in all but one case, so the risk to benefit ratio of corticosteroid administration should be considered on an individual basis.

Advances in treatment for trigeminal‐mediated headshaking will remain limited until the aetiopathogenesis of the condition is understood. It is certainly possible that there is more than one cause with the same clinical manifestation, leading to different response rates to treatment. It is recognised in human medicine that response to treatment for neuropathic pain varies amongst individuals, even with the same diagnosis 14. Therefore, even if all trigeminal‐mediated headshakers have the same underlying condition, response to the same treatment may vary. Also, the mechanism of action of any neuromodulatory therapy is not well understood. There is a need for research into neurophysiology pre‐ and post‐neuromodulation in any species. The lack of knowledge in this area limits our ability to determine whether adaptations of the EquiPENS™ technique could lead to remission in non‐responders and longer remission times in responders. It also limits our ability to compare the technique to electroacupuncture 11, leaving the only way to make a comparison being to compare the use of a uniform electroacupuncture technique over a similar number of appropriately diagnosed horses.

In conclusion, based on these results, we recommend that at present, EquiPENS™ neuromodulation be considered for the management of trigeminal‐mediated headshaking in horses where a nose‐net is unsuitable. Further advances in the understanding of the aetiopathogenesis of trigeminal‐mediated headshaking are vital for there to be real progress in treating the condition. In the shorter term, a greater understanding of neuromodulation could aid refinement of the EquiPENS™ technique.

## Authors’ declaration of interests

The University of Bristol receives a fee for training centres in the procedure, part used to cover professional time and part used to fund research into headshaking.

## Ethical animal research

The study received University of Bristol ethics approval, registered with Veterinary Investigation Number 18/011.

## Owner informed consent

Informed consent was obtained from horse‐owners.

## Source of funding

The Academy of Medical Sciences and the Wellcome Trust’s Inspire Research Studentship funded a 1‐month student post to help with data collection.

## Authorship

V. Roberts contributed to study design, study execution, data analysis and interpretation, and preparation of the manuscript. N. Patel contributed to study design, and data analysis and interpretation. M. Bailey contributed to study design, and data analysis and interpretation. The EquiPENS group contributed to study execution and preparation of the manuscript. All authors gave their final approval of the manuscript.

## Supporting information


**Supplementary item 1** **:** Collaborators and affiliations within the EquiPENS™ Group.

**Supplementary item 2** **: **Guidelines issued to centres for diagnosis.

**Supplementary item 3** **: **Data collected from owners at follow‐up.

**Supplementary item 4** **: **Distribution of age (years) at first presentation of horses in each group of breed types.

**Supplementary item 5** **: **Distribution of number of cases across the centres.

**Supplementary item 6** **: **Uses of horses.

**Supplementary item 7** **: **Main treatments attempted prior to receiving neuromodulation.

**Supplementary item 8** **: **Less commonly used, attempted but failed treatments.

## Manufacturers' addresses

1 Algotec Reseach and Development UK, Crawley, West Sussex, UK.

2 The R Project for Statistical Computing, Vienna, Austria.

3 Canonical Ltd, London, UK.
