# Supplementary material for: The safety and efficacy of neuromodulation using percutaneous electrical nerve stimulation for the management of trigeminal‐mediated headshaking in 168 horses
Source: Equine Vet J. 2019 Sep 23;52(2):238–43. doi: 10.1111/evj.13174 (PMC7317358; doi:10.1111/evj.13174)
Supplement: Supplementary file 1 — Supplementary item 1 : Collaborators and affiliations within the EquiPENS™ Group. [file EVJ-52-238-s001.pdf]

BEVA Equine Veterinary Journal

Supplementary Item 1:

Collaborators and affiliations within the EquiPENS™ Group

H. B. Carslake[1], F. Malalana[1], C. McGowan[1], E. F. Haggett[2], T. Barnett[2], D. I. Rendle[3], U. Lunden[4], A. R. Fiske-Jackson[5], V. E. South[6], J. Prutton[6], A. Durham[6], R. Findshøj[7], H. Panhuijzen[8], R. Van Der Rijt[9], T. Booth[10], M. Robin[11], A. G. Raftery[12] and K. J. Pickles[13]

1. The Philip Leverhulme Equine Hospital, Institute of Veterinary Science, University of Liverpool, Leahurst Campus, Chester High Road, Neston, Wirral, CH64 7TE, UK.

2. Rossdales Equine Hospital, Cotton End Road, Exning, Newmarket, Suffolk, CB8 7NN, UK.

3. Rainbow Equine Hospital, Rainbow Farm, Malton, North Yorkshire, YO17 6SG, UK.

4. Evidensia Strömsholm, Djursjukhusvägen 11, 734 94 Strömsholm, Sweden.

5. Royal Veterinary College, Hawkshead Lane, Hatfield, Hertfordshire, AL9 7TA, UK.

6. Liphook Equine Hospital, Forest Mere, Liphook, Hampshire, GU30 7JG, UK.

7. Evidensia Faxe Dyrehospital, Industriparken 11 4640, Faxe, Denmark.

8. DAP Bodegraven, Zuidzijde 63, Bodegraven 2411 RT, Netherlands.

9. Pool House Equine Clinic, Crown Inn Farm, Rykneld Street, Fradley, Lichfield, Staffordshire, WS13 8RD, UK.

10. Shelf Equine Clinic, Giles Hill Lane, Shelf, Halifax, West Yorkshire, HX3 7TW, UK.

11. Fyrnwy Equine Clinic, Whitmore Lane, Baschurch, Shrewsbury, Shropshire, SY4 2EY, UK.

12. Weipers Equine Centre, University of Glasgow, Glasgow, G61 1QH, UK.

13. Chine House Equine Hospital, 12 Cossington Road, Sileby, Leicestershire, LE12 7RS, UK.
