# Supplementary material for: The safety and efficacy of neuromodulation using percutaneous electrical nerve stimulation for the management of trigeminal‐mediated headshaking in 168 horses
Source: Equine Vet J. 2019 Sep 23;52(2):238–43. doi: 10.1111/evj.13174 (PMC7317358; doi:10.1111/evj.13174)
Supplement: Supplementary file 2 — Supplementary item 2 : Guidelines issued to centres for diagnosis. [file EVJ-52-238-s002.pdf]

**Supplementary Item 2: Guidelines issued to centres for diagnosis.**

Horses should have been diagnosed with trigeminal-mediated headshaking.

**i. Gold standard:**

The gold standard for this is likely to be proof that the trigeminal nerve is sensitised, obtained by performing somatosensory-evoked potentials. However, these must be performed under general anaesthesia.

**ii. History, signalment, observation:**

History, signalment and observation are likely to give you your index of suspicion for trigeminal-mediated headshaking.
Headshaking is often acute in onset although some cases will be insidious in onset. Onset typically occurs between the ages of 5 and 12 years. About one quarter of trigeminal-mediated headshakers will improve when wearing a nose-net, so improvement with a nose-net is consistent with trigeminal-mediated headshaking, although a failure to improve with a nose-net does not rule out this diagnosis. I would not expect trigeminal-mediated headshakers to respond to administration of phenylbutazone or systemic or inhaled corticosteroids. They might respond to medication such as carbemazapine or gabapentin.
Headshaking is usually predominantly vertical and often accompanied by sharp, vertical twitches. There are usually signs of nasal irritation, such as snorting, rubbing the nose on surfaces or legs and sometimes striking at the nose. Signs may be seen at rest in the stable and or field, but are usually worse at exercise. Although they may be worse when ridden than on the lunge, signs would usually be seen at exercise without a rider. About one third of cases are seasonally affected, and if so these are usually Spring/Summer affected. Some horses will show signs when outside, but not inside, on the same day.

**iii. Are signs of headshaking due to facial pain?**

If possible, demonstrate that the horse is headshaking due to facial pain. This would be demonstrated if the horse shows a positive response to diagnostic local anaesthesia of the trigeminal nerve. Only two sections of this nerve are practically accessible for infiltration of local anaesthetic. The most rostral part of the infraorbital nerve may be anaesthetised, which is best accessed as far caudally as possible as it emerges at the infraorbital foramen. It is relatively simple to perform but there is evidence to suggest that response is usually poor. The poor response may be that only a small portion of the nerve is affected by the local anaesthetic in this position. Trigeminal-mediated headshakers usually show signs of nasal irritation. Innervation to the nasal mucosa is from the caudal nasal nerve, a branch of the infraorbital nerve which runs parallel to this nerve before branching in the caudal nasal foramen, usually caudal to the rostral aspect of teeth 109/209. Infiltration of local anaesthetic at the infraorbital foramen would not involve the caudal nasal branch and involvement of this branch may be important. It is perhaps because of this that results from diagnostic local anaesthesia of the caudal portion of the infraorbital nerve, before it enters the infraorbital canal, are better. A positive response to diagnostic local anaesthesia of the infraorbital nerve would be consistent with headshaking due to facial pain, although a negative result would not refute it as there are many limitations to the technique.

**iv. Is there gross pathology which could be responsible for causing facial pain?**

The next stage is to look for any gross pathology which could be responsible for the facial pain. Where an abnormality is found, it could be treated and then the horse observed to see if signs of headshaking are resolved, as abnormalities may be incidental. I perform upper respiratory tract and guttural pouch endoscopy, oral examination, basic ophthalmic examination. Where possible, computed tomography of the head should be performed and, if not, radiographs of the head performed.

**v. Grade the severity of headshaking**

This may be graded as:
0/3 = no headshaking;
1/3 = headshaking at exercise but insufficiently severe as to interfere with ridden exercise;
2/3 = headshaking at exercise, of a severity sufficient to make riding impossible or dangerous;
3/3 = headshaking even at rest, in the stable and/or field.

Please contact me to discuss suitability for treatment, if you have any cases which do not meet these diagnostic criteria.
