# Supplementary material for: The safety and efficacy of neuromodulation using percutaneous electrical nerve stimulation for the management of trigeminal‐mediated headshaking in 168 horses
Source: Equine Vet J. 2019 Sep 23;52(2):238–43. doi: 10.1111/evj.13174 (PMC7317358; doi:10.1111/evj.13174)
Supplement: Supplementary file 3 — Supplementary item 3 : Data collected from owners at follow‐up. [file EVJ-52-238-s003.pdf]

BEVA Equine Veterinary Journal

Supplementary Item 3: Data collected from owners at follow-up.

Centres were asked to complete the following information during telephone follow-up to owners:

Number of procedure	Date	Complications? If so, details	Remission (back to ridden work at previous level or above)?	Length of remission	Date of last follow-up
1			Yes / No		
2			Yes / No		
3			Yes / No		
4			Yes / No		
5			Yes / No		
6			Yes / No		
