# Supplementary figures and images for: The safety and efficacy of neuromodulation using percutaneous electrical nerve stimulation for the management of trigeminal‐mediated headshaking in 168 horses
Source: Equine Vet J. 2019 Sep 23;52(2):238–43. doi: 10.1111/evj.13174 (PMC7317358; doi:10.1111/evj.13174)

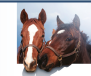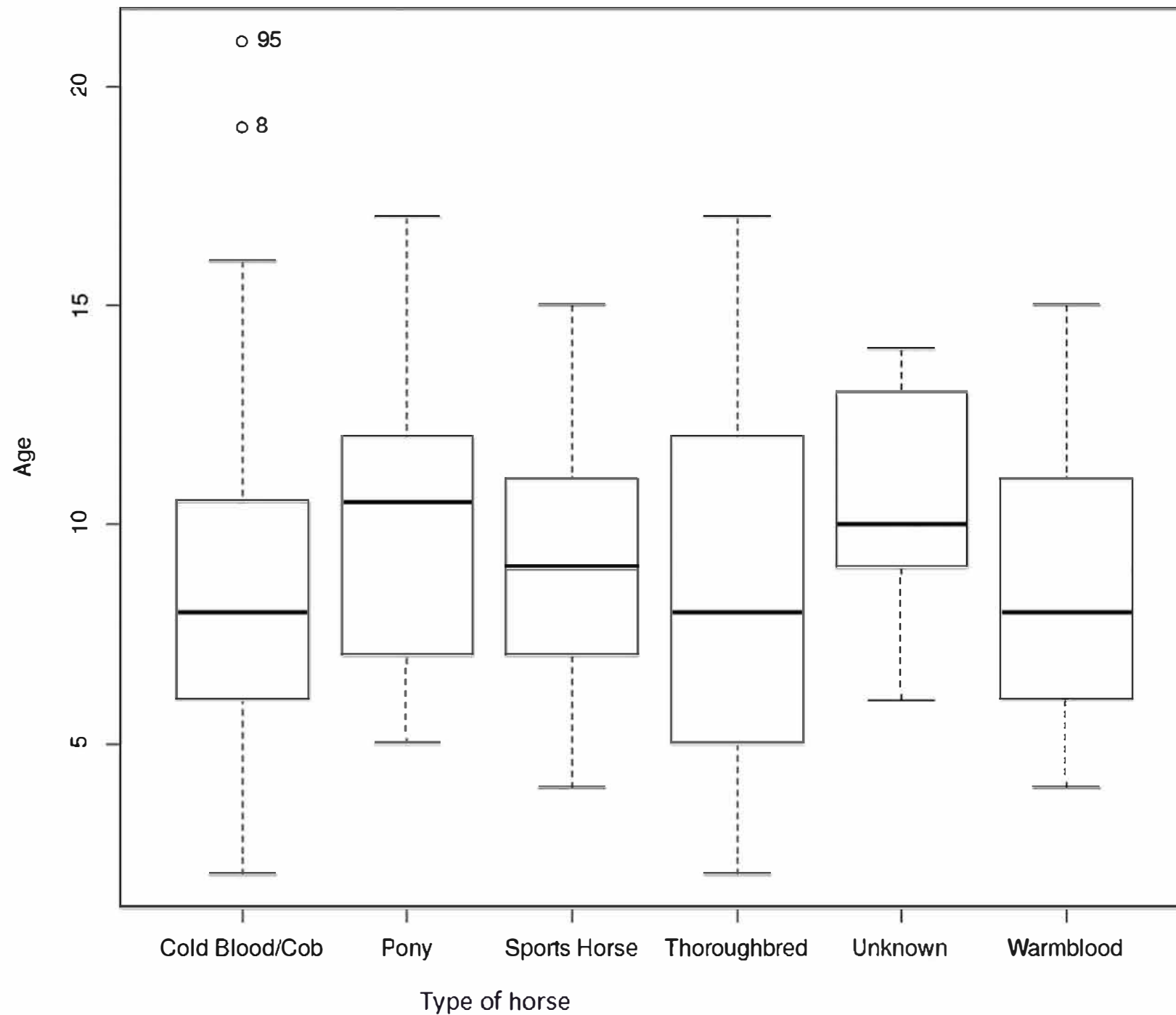

Supplement: Supplementary file 4 — Supplementary item 4 : Distribution of age (years) at first presentation of horses in each group of breed types. [file EVJ-52-238-s004.pdf]

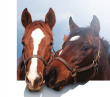

**Supplementary Item 5:** Distribution of number of cases across the centres.

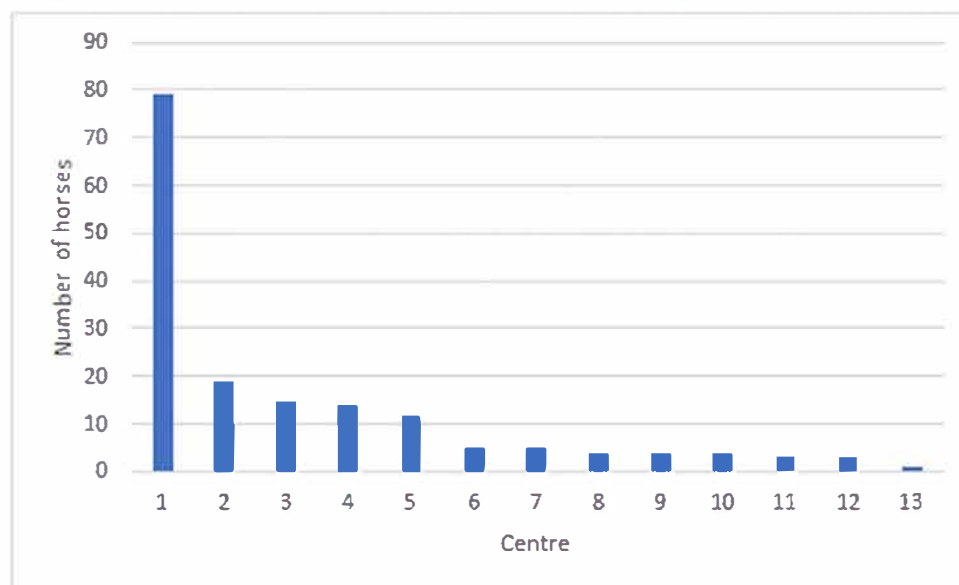

Supplement: Supplementary file 5 — Supplementary item 5 : Distribution of number of cases across the centres. [file EVJ-52-238-s005.pdf]

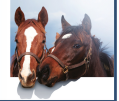
**Supplementary Item 6: Use of horses.**
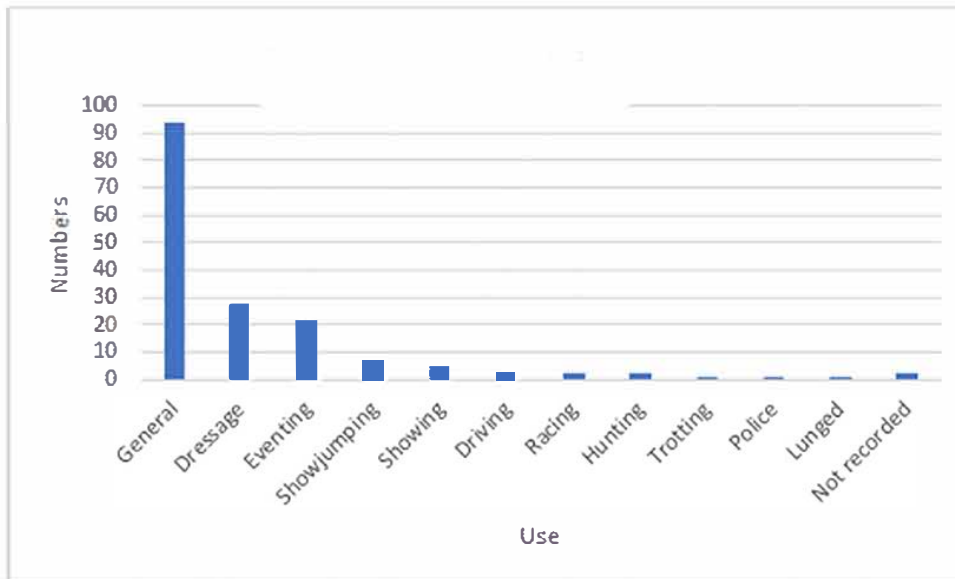

Supplement: Supplementary file 6 — Supplementary item 6 : Uses of horses. [file EVJ-52-238-s006.pdf]

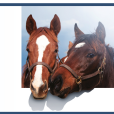

## Supplementary Item 7: Main treatments attempted prior to receiving neuromodulation.

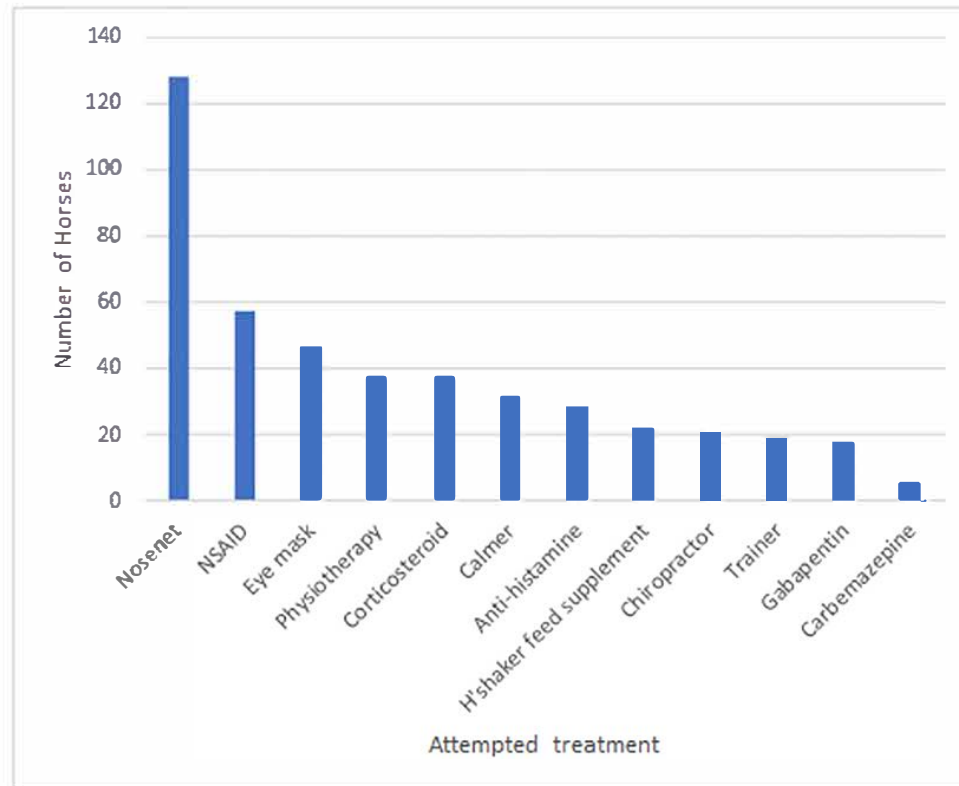

Supplement: Supplementary file 7 — Supplementary item 7 : Main treatments attempted prior to receiving neuromodulation. [file EVJ-52-238-s007.pdf]
