# Supplementary material for: The safety and efficacy of neuromodulation using percutaneous electrical nerve stimulation for the management of trigeminal‐mediated headshaking in 168 horses
Source: Equine Vet J. 2019 Sep 23;52(2):238–43. doi: 10.1111/evj.13174 (PMC7317358; doi:10.1111/evj.13174)
Supplement: Supplementary file 8 — Supplementary item 8 : Less commonly used, attempted but failed treatments. [file EVJ-52-238-s008.pdf]

BEVA Equine Veterinary Journal

Supplementary Item 8: Less commonly used, attempted but failed treatments.

TREATMENT	NUMBERS OF HORSES
'Micklem'[1] bridle or similar	7
Acupuncture	6
Bitless bridle	6
Cellulose based nostril powder spray 'Nostril Vet'[2]	3
Application of petroleum jelly to the inside of the nostrils	3
Medication of the temporomandibular joint with corticosteroid	3
Ear covers	3
Salt supplementation	2
Cranial therapy	2
Application of magnets to the headcollar	2
Omeprazole	1
Suspension of powdered Sarracenia purpurea (Sarapin[3]) injected subcutaneously at the infra-orbital foramina	1
Acepromazine[4]	1
Valerian	1
Melatonin	1
Feeding spirulina	1
Feeding a low starch diet	1
Taurine supplementation	1
Allergen immunotherapy	1
Fly repellent	1
Ceratohyoidectomy	1
Extraction of the first pre-molars	1
Extraction of a molar and supranumerary tooth	1
Diastema treatment	1
Medication of the nuchal bursa with corticosteroid	1
Laser treatment	1
Ultrasound treatment	1
Sinus lavage	1
Riding in the dark	1
Riding in a headcollar	1
Padding the poll under the bridle	1
Bucket nose muzzle	1

Manufacturers' addresses

[1]William Micklem, Annacrivey Stud, Enniskerry, Co. Wicklow, Ireland.

[2]Kisska International Ltd, Nasaleze International Ltd, Douglas, Isle of Man, UK.

[3]High Chemical Company, Levittown, Pennsylvania, USA.

[4]Vetoquinol UK Ltd, Towcester, Northamptonshire, UK.
